# Insecticides for Mosquito Control: Improving and Validating Methods to Strengthen the Evidence Base

**DOI:** 10.3390/insects14020116

**Published:** 2023-01-23

**Authors:** Rosemary Susan Lees, Christen Fornadel, Janneke Snetselaar, Joe Wagman, Angus Spiers

**Affiliations:** 1 Department of Vector Biology, Liverpool School of Tropical Medicine, Pembroke Place, Liverpool L3 5QA, UK; 2 Innovation to Impact, Liverpool School of Tropical Medicine, Pembroke Place, Liverpool L3 5QA, UK; 3 Innovative Vector Control Consortium (IVCC), Liverpool School of Tropical Medicine, Liverpool L3 5QA, UK; 4 PATH, 455 Massachusetts Ave NW, Washington, DC 20001, USA

## 1. Background: Good Decisions Require Good Data

Efforts to eliminate vector-borne diseases, for example malaria which caused an estimated 619,000 deaths in 2021 [1] or arboviral diseases such as dengue and zika [2], rely heavily on the use of vector control tools. The toolbox available to combat insect vectors of disease is growing through improvements to existing approaches and new, emerging, technologies. New chemistries are being developed to target pyrethroid-resistant malaria vectors, for use in conventional tools such as insecticide-treated nets (ITNs) and indoor residual sprays (IRS), as well as through innovative means of deployment such as attractive targeted sugar baits (ATSB), passive emanators and eave tubes. Rear and release strategies to control *Aedes* vectors of arboviruses are under pilot evaluation, including versions of the sterile insect technique (SIT) and the use of *Wolbachia* symbionts for population control or replacement. These tools are also being piloted to urgently combat the expansion of *Anopheles stephensi* in Africa.

The decision to deploy new vector control tools or approaches on an operational level should be supported by robust entomological evidence to demonstrate efficacy, comprising data collected using appropriate and validated methods. A strong evidence base can also guide effective operational deployment decisions. The Insects Special Issue “Insecticides for Mosquito Control: Strengthening the Evidence Base” presents original research into developing and characterising new vector control products, as well as understanding and monitoring insecticide resistance. Review articles explore the impact of insecticide resistance and offer guidance on insecticide choice in the face of pyrethroid resistance. Consensus methodologies are presented, in the form of standard operating procedures (SOPs) designed to be adopted and used to generate reproducible data that can be compared and interpreted across and between studies. It is hoped that this Special Issue offers inspiration and guidance on how consistent data can be generated to inform more effective development, evaluation and use of new and existing vector control tools. 

## 2. The Impetus to Better Validate Entomological Methods

Issues around improved generation and interpretation of entomological data are particularly timely in light of the establishment in 2016 and ongoing evolution of the World Health Organization (WHO) Prequalification Vector Control Team (PQ/VCT), whose mandate is to “increase access to safe, high-quality and effective VCPs” (vector control products) by working to evaluate and prequalify tools and contribute to building assessment capacity in national regulatory authorities. Prequalification streamlines access to vector control products by employing regulatory best practices to product evaluation and provides relevant information to help guide decisions about procurement and implementation. However, robust decisions can only be made using high quality, consistent data. 

Implementation of good laboratory practice (GLP) compliance in an international network of vector control testing facilities was an important first step to improving quality of entomological data [3]. GLP compliance offers reliable, auditable, and reproducible data, but does not guide on testing methodology. Standard protocols are available in WHO guidance and elsewhere for evaluating the bioefficacy of vector control tools, but these were developed primarily to measure the fast-acting lethal effect of pyrethroids in contact assays and may not be appropriate for new modes of action or methods of active ingredient (AI) delivery. In order to spur innovation, it is crucial that validated methods are available for tools with different modes of action to measure the relevant end points with sufficient accuracy, sensitivity and reproducibility. 

This Editorial will consider in detail the issues relating to entomological efficacy testing of insecticides and insecticide-based products targeting adult *Anopheles* mosquitoes, with a particular focus on ITNs and IRS. However, the illustrations of good practice and recommendations highlighted here are more widely applicable to other types of tools, as evidenced by many of the articles included in the Special Issue [4,5,6]. This includes tools targeting *Aedes* mosquitoes [7] where the commercial market is consumer-driven, the regulatory framework is more flexible and the guidance on evaluation sparser. There are gaps in the guidance for the evaluation of larvicides and spatial repellents, for both *Aedes* and *Anopheles* control. Many issues highlighted in this discussion will also be relevant for the collection and interpretation of epidemiological data relating to the efficacy of vector control tools, and to the need to better characterise the mode of action and impact of new tools [8,9].

The most widely used methods for measuring the insecticidal bioefficacy (i.e., the ability of the insecticide component(s) of a vector control tool to kill susceptible target vectors) of malaria vector control tools include cone bioassays, tunnel tests, and experimental hut trials (EHTs) [10]. Cone bioassays have been used successfully to evaluate and monitor ITN and IRS bioefficacy for more than 20 years and have proven able to generate GLP-quality data for two important, rapidly induced, easy to measure outcomes: mosquito knockdown and mortality. These outcomes, which are usually measured around 60 min after exposure for knockdown and 24 h after exposure for mortality, are critically important factors that influence how effective a vector control tool can be at preventing disease transmission and are, therefore, important to monitor over time. However, these knockdown and mortality endpoints, similar to the cone bioassay initially implemented to measure them, were designed specifically to easily test the immediate effects of fast-acting, topically exposed neurotoxins such as DDT and pyrethroids. They were never intended, or adequate, for capturing the full range of insecticidal modes of action on vector biology or disease transmission. 

These shortcomings led to the inclusion of additional outcomes that are typically measured with tunnel tests and EHTs, and measures such as blood-feeding inhibition, deterrence, induced exophily, and delayed mortality that have become more widely used [11]. Tunnel tests and EHTs have proven useful for monitoring the efficacy, and predicting the effectiveness of, vector control products over time and for guiding product development. However, their outcome measures have proven more difficult to standardize, replicate, and interpret across a diverse range of research and implementation settings. These difficulties are being exacerbated by the arrival to market of new active ingredients (AIs) with novel modes of action–for example, the delayed mortality induced conditionally by chlorfenapyr [11] and others and the reduced mosquito fecundity induced by pyriproxyfen [12]. Additionally, complicating the testing landscape are new tools that combine insecticides from different insecticidal classes with different modes of action, whose bioefficacy needs to be evaluated independently using multiple mosquito strains and/or endpoints [13], and novel interventions that utilize alternative routes of insecticide delivery distinct from the tarsal exposure to ITNs and IRS, for example the ingestion of insecticidal AIs facilitated by ATSBs [4].

## 3. The Need for New and Improved Methods

If we are to properly understand the entomological effects of new products and evaluate them robustly, we need to use well-validated methods. Using methods which are fit for purpose is crucial to enable regulators, procurers and implementers to assess and compare the range of tools available to them in order to make informed decisions on how best to utilise limited operational budgets. Clearly, innovation in testing methodology is needed so that testing outcomes describe all the important aspects of a product’s insecticidal efficacy but avoid collecting extraneous information or data that is ultimately tangential to distinct product claims. Without such methods, it is difficult to appropriately monitor the entomological effects of a new tool deployed at scale. This becomes especially important in situations where the introduction of a new vector control tool does not have the expected epidemiological impact–without measuring the appropriate entomological efficacy endpoints using the best available methods it will not be possible to elucidate why this might be. For new products in development, it is important to understand not only *that* a product class has an impact, but also *how* it achieves this impact. 

Standardised and/or characterised inputs will help to reduce methodological error and variability in the data and help with interpretation of results. One key input is the insects used for testing. The insecticide resistance profiles of the various mosquito strains used for testing should be characterised, be they ‘susceptible’ or ‘resistant’ strains [14,15,16]. The cohorts of mosquitoes used for testing should be generated using standardised rearing, and fitness parameters such as size recorded [14]. In addition to the insects, it is important that the key testing parameters should be standardised or characterised as far as possible, for example using tools such as the Micron Track Sprayer to improve consistency of insecticide application [17], by interrogating methods to identify and minimize possible sources of variability [18], and by optimising methods to improve the consistency of the data [19]. Standardised methodologies such as SOPs generated by consensus [13,14] will facilitate a comparison and interpretation of the results between testing sites and across studies. Data collection should be made as objective as possible, for example by the use of automated scoring tools [20]. It is also best practice, when reporting results of a study, to include methodological detail alongside the data and ensure that raw disaggregated data, including control data, is presented.

We rely on data from efficacy and insecticide resistance testing to make choices about product use and understand the cross-resistance risk for different AIs. However, even with pyrethroids, there is ambiguity in the data generated by accepted methods and a need to understand the sources of variability and characterise or standardise inputs. For example, there is insufficient evidence from testing data or our understanding of the mode of action to confidently recommend the rotation of different pyrethroids for resistance management [21]. 

The establishment of the WHO PQ/VCT has changed the way in which products are evaluated by WHO, bringing these processes more in line with best practice regulatory approaches and offering a significant opportunity for the robust evaluation of new product classes. PQ/VCT’s approach is flexible, allowing applicants to agree methods with WHO that best reflect their product’s mode of action, rather than adhering to rigid, standard methodologies. This process still requires any data generated to be robust, consistent and appropriate to the product and its specific mode of action and claims. It is a significant step forward to support innovation in vector control; however, the approach has highlighted long standing issues in the generation and interpretation of entomological data to evaluate new tools. Investment in validating methods to generate robust entomological data has been lacking, meaning that potentially effective tools are unable to smoothly progress through regulatory processes or be consistently evaluated in the field. Developing and validating methods that are fit for purpose will help to streamline decision-making processes by better defining the effect of a tool and reducing the need for the generation of additional supporting data. 

Beyond introduction to the market, there is a need to manage the lifecycle of a product, for example determining whether an ITN is performing as expected throughout its 3-year lifespan. If effective methods are not available to measure a product’s performance during its active life, it is difficult to measure the appropriateness for implementation in a given context. Entomological data are important for monitoring durability of ITNs, but it should be supported by chemical analysis of the total AI content, or ideally the surface content and presentation of AI [22]. Improvements and adaptations to new product types are also important for analytical methods [23].

## 4. Relating Results of Laboratory and Semi-Field Tests to Product Performance

It is important that there is clarity on what entomological endpoints should be measured in laboratory, semi-field and field experiments, in order to inform our understanding of how a vector control product will perform. Taking ITNs as an example, efficacy is described in the new WHO Guideline for the Prequalification Assessment of Insecticide-Treated Nets [22] as being influenced by potency, biologically available fraction of the surface concentration of AI, net construction, uptake of AI by free-flying target vectors, as well as handling and care of the ITN. Bioassays in the laboratory can be used to ascertain the efficacy of a product against lab strains and to some extent wild populations under controlled conditions, giving a measure of surface available insecticide across a net and its potency through uptake of this fraction. Semi-field bioassays can provide additional measures of uptake of insecticide by free-flying mosquitoes under more ‘real life’ conditions. These parameters predict effectiveness, or how well the net may perform in the real world in terms of entomological and, potentially, epidemiological outcomes [24]. 

Such sequential testing using increasingly sophisticated methods has been the accepted approach to determining efficacy and predicting effectiveness. The new WHO PQ/VCT approach, however, allows more flexibility, facilitating, for example, the progression of slower-acting toxicants and pro-insecticides such as chlorfenapyr. Standard lab bioassays were developed to measure the effect of fast-acting pyrethroids, and measure endpoints which are not appropriate for a pro-insecticide. Unlike pyrethroids that kill by acute neurotoxicity, chlorfenapyr kills by disrupting a mosquito’s ability to produce energy in the mitochondria, after it has first been metabolised into its active forms. The more physiologically active a mosquito, the greater the likelihood of higher conversion rates to these active forms. This process of conversion is also enhanced by biochemical processes such as metabolic activity of P450 enzymes. Thus the metabolic state of the mosquito is extremely important during testing. As a result, chlorfenapyr-treated ITNs perform poorly in the lab under artificially controlled testing conditions using standard methods [25], but better in semi-field testing [26,27], and have been shown to have a significant epidemiological impact [28]. This example illustrates the need to use appropriate and validated methods to evaluate a given product or product type, and to avoid the over-interpretation of entomological data.

The results of bioassays and semi-field studies should be interpreted with caution as a prediction of performance of an ITN, defined by WHO PQ/VCT as its ability to provide continuous controlled release of insecticide and maintain physical integrity under normal use [22]. The link between bioassay data and entomological or epidemiological impact when a product is deployed is even less clear, and results should not be conflated to make implementation decisions. To take the ITN example, durability monitoring should measure the effective life of an ITN whereby bioassays are a proxy for surface AI availability and should not be conflated with effectiveness. Laboratory washing methods are used to measure regeneration time and as a means to artificially age nets, but they may not reflect the treatment of nets under normal use conditions [29]. In IRS testing, the walls of experimental huts treated with test products to measure residual efficacy may not accurately represent the results of real-world application, though new methods and equipment can at least be used to improve accuracy of wall treatment [17]. Methods should be chosen or designed to accurately measure the intended entomological endpoint for a given purpose, and we must be consistent about how the data are interpreted and careful not to conflate results from testing carried out for different purposes or measuring different endpoints.

Monitoring of insecticide resistance in target vector populations is another example of bioassay data being used beyond the scope of questions methods were designed to answer. Using a discriminating concentration to test mortality in wild caught mosquitoes should be routine practice to monitor for emergence of resistance in a vector population as a warning sign that a product may start to fail [30]. The WHO cylinder and bottle tests were designed to provide information about intrinsic susceptibility to the insecticide, not as a predictor of product efficacy, or to predict product effectiveness at a given location. Further, susceptibility testing methods may need to be adapted to consider different means of deploying insecticides, for example ingested insecticide in attractive targeted sugar bait (ATSB) products [4].

We are left with the question of how far we might be able to link entomological end points, measured through bioassays, and product impact. Better understanding of existing testing methods and how to use the data they generate will be key. For example, EHTs start to bridge the gap between cone tests and entomological impact and are the gold standard, but the link between EHT results and resistance is highly uncertain [31]. Recent analysis shows that EHT data can be used to parameterise models and reliably predict epidemiological impact of rapid-acting pyrethroids on ITNs and IRS [24]. Conversely, modelling may be used to more meaningfully interpret and use the data generated by laboratory and semi-field bioassays. Additional data may be generated while applying existing methods, for example by measuring not just knock down or mortality after exposure to an insecticide-treated product but also measuring sublethal effects of exposure [8]. Delayed mortality, reduced fecundity, reduced blood feeding and other parameters may result in entomological impact. It is important to observe end points that are relevant to the mode of action and intended effect of the product under evaluation, which relies on understanding the wholistic impact of insecticide exposure on mosquito populations [9]. Sublethal effects of insecticide exposure are much more important to understand in the case of highly resistant populations, and slow-acting mortality is important to measure and understand for different modes of action. 

If we were able to more directly connect the results of small-scale entomological experiments, enhanced by using a range of well-characterised vector colonies to reflect a wide range of target populations [16], to entomological and ultimately epidemiological impact, then we could potentially reduce the need for costly and lengthy clinical trials and speed the route marketing new products. This has been one aim of the New Nets Project, which is implemented by a consortium of partners led by the Innovative Vector Control Consortium (IVCC) to build the evidence needed to influence policy in this area [32] through enhanced data collection during randomised control trials and under operational pilot conditions. In some cases, it may be necessary to develop new methods to collect the evidence that is needed. An example is provided by the Ifakara Ambient Chamber Test (I-ACT) [33], which allows more controlled and high throughput evaluation of the efficacy of vector control products in semi-field conditions and provides greater statistical power than an EHT; thus, it is an important additional method.

## 5. The Need for Pre-Emptive Method Development and Validation

For vector control products with novel modes of action that are considered “first in class,” the WHO Vector Control Advisory Group (VCAG) requires two epidemiological trials to demonstrate public health value before a product class can be recommended and a “first in class” product can receive a WHO prequalification listing. Any subsequent product that elicits a similar entomological effect should be able to receive a policy recommendation based on accepted and validated entomological methods with well understood links to epidemiological outcomes. This has been demonstrated for pyrethroid-only nets, where EHTs predict performance well [24], and a similar analysis is underway to correlate hut trial results for chlorfenapyr-containing nets with field performance. However, when considering lab-based assays, any correlation for chlorfenapyr is challenging because commonly used methods do not capture its mode of action effectively. When undertaking efficacy testing for novel vector control chemistries/products, it is important to understand not just what the entomological effect is, but also how that effect is produced.

By using appropriate methods, that are validated before a product is brought to market, bottlenecks to access are reduced and new tools can be adopted with higher confidence in their performance. Additionally, there may be scope to improve products after launch if we understood them better, for example by selecting a more active crystalline form of an insecticide to use in IRS [34]. A good example of a testing pipeline for a novel insecticide from mode of action to method of deployment is described by Mysore et al. [5]. Innovative new approaches need clear guidance on the data required to demonstrate efficacy and the methods to collect them. Plant-based compounds [6] or RNAi approaches [5], for example, may be used to circumvent resistance, but it’s important to ensure that WHO guidelines for efficacy and resistance testing are appropriate for these alternative AIs and tools. Similarly, efficacy testing of products developed without insecticides such as bite-resistant fabrics [7] are not covered by current guidelines or standard methodologies.

There is a need for a broad and robust data set before a new product is brought to market, which relies on having robust methods to measure entomological endpoints, as well as solid interpretation, analysis and use of the data generated. WHO PQ/VCT would ideally be evaluating products using data generated with validated methods, but thus far no guidance is available as to what method validation should consist of, beyond the Collaborative International Pesticide Analytical Council (CIPAC) methodologies which focus on analytical chemistry [35]. CIPAC presents a clear framework for validation; however, it is a challenge to apply analytical standards to bioassays as they are not realistic for a biological system. There is a need for rigorous standards and validation of methods, but until recently there has no guidance on how to perform validation of entomological methods [36]. This gap has resulted in products going into use before there are established methods for durability or resistance monitoring, whereas is it important that sufficient baseline data is collected to monitor for loss of efficacy with use or reduced susceptibility as an insecticide-based product is deployed [4]. For some products this could mean that there is already unidentified cross-resistance in the target population. In some cases, the only methods available for their evaluation can only be performed in certain sites, for example the tunnel test currently relies on access to an animal host to perform well [19]. In other instances, it will be important to understand possible interactions between products used in integrated vector management, and those methods also need to be developed [37].

## 6. Conclusions: A Timely Opportunity to Drive Improvement in Method Validation and Evidence Generation 

The establishment of the WHO PQ/VCT process, and the welcome focus on a regulatory approach to product evaluation, offers the opportunity for a fundamental change in the way we view vector control products. The promise of this new process is that product developers can generate data that reflects the performance of their product, rather than developing products to meet a rigid set of data requirements. There is an opportunity to move away from thresholds applied to bioassay results and used to judge efficacy of products, and towards a more rounded “weight of evidence” evaluation which allows a greater understanding of how a specific product works, making it easier to compare it to other classes and meaningfully monitor ongoing efficiency. This in turn should help to correlate entomological endpoints with product efficacy and impact, and will allow more informed procurement and deployment decisions. However, this flexibility must be underpinned by robust, reproducible data that clearly support product claims that are independently validated. The fact that many current entomological methods have been designed to measure the rapid kill and knockdown of pyrethroids hinders the exploration of different modes of action or methods of delivering AIs. As such, investing in methodological development is key to help spur future innovation in vector control. 

Validation of methods is crucial to ensure that results are reproducible and informative. Even seemingly standard methods, such as the WHO tube test, show significant variation if testing is not standardised [18]. Innovation can be applied to improve data generation even with standard methods [19]. Taking steps to utilise methods that accurately detect relevant entomological endpoints will be crucial to interpreting and comparing new tools with different modes of action. This will be complicated by the wider range of entomological effects induced by new AIs, and the relative impact of the same level of, for example, sterilisation or delayed action mortality may not be obvious. Data should be generated using validated methods that are characterised in terms of the natural variability of data and more effort must be made to characterise material inputs to provide further context to results. The responsibility for method development and validation for product evaluation and monitoring lies primarily with manufacturers, who understand their products best, with the support from the research community on innovation and development. There is a potential role for an independent body tasked with the validation of proposed entomological methods on behalf of the manufacturers, analogous to the role of CIPAC for methods in analytical chemistry.

Currently, data generation for vector control tools is centered around access to the market with a focus on a WHO policy recommendation and WHO PQ/VCT listing. These are important milestones, but data is needed throughout a product’s lifecycle to inform on performance and aid deployment decisions, particularly when implementing resistance management strategies. Recent history has seen the development of lifecycle management methods only occurring post market, meaning products in use may not have reliable methods to generate data on performance trends and ascertain resistance issues when first launched. We therefore recommend that a comprehensive package of data is generated for a vector control product that goes beyond market access and encompasses lifecycle management. This should include a package of validated methods for generation of the data throughout a product’s lifecycle, and a means for interpretation to assist decision making for implementation. Some of this information will be available through established evaluations (e.g., WHO PQ/VCT), but others should be considered in addition to those requirements to ensure streamlined uptake of new tools.

- **Scope of the product:** detailed description of, for example, under what conditions it is expected to be effective, the target species, and what resistance mechanisms exist in the target population/s which might be relevant.
- **Entomological mode of action**: as detailed a description as possible about how the product acts on the target species to elicit the intended effect, which may include IRAC classification [38] and is important to the understanding of cross-resistance risks and potential interaction between products.
- **Intended entomological endpoints:** clear definitions of the entomological effects which are relevant to the product and should be measured to demonstrate efficacy.
- **Regeneration time:** clear understanding of the dynamic presence of insecticide within the product or its sphere of influence, for example the time taken for AI to regenerate on the surface of an ITN after washing.
- **Insecticide content and formulation:**
  - The functionality of an insecticide-based product depends on the amount of insecticide present (a) in total, (b) on the surface, and (c) in the bioavailable fraction which vectors are able to pick up. Insecticide content needs to be monitored throughout the life cycle of a product.
  - Knowing the way that a product is formulated and the insecticide is presented to the mosquito is also critical to understanding how a product works, for example whether an ITN is coated with an AI or the AI is incorporated into the fabric, or whether an IRS formulation is a suspension or microencapsulated formulation. The presentation of AI may change over the life of the product.
  - An understanding of both insecticide content and presentation is needed in two settings:
    1. Under standard conditions in the laboratory, where, for example, it may be sufficient to use analytical methods to measure AI concentration since the aim is to understand the properties of the product and monitor quality.
    2. Under real world conditions, where the aim is to understand a product’s effectiveness and so bioassays should be used in place of, or to confirm, analytical methods.
- **Residual efficacy**: three elements of a product should be monitored over time:
  - **Bioefficacy**, or the ability of the product to elicit the intended entomological effect. This may be measured through a bioassay as a proxy measure for the bioavailable fraction of AI, or an analytical method which has been shown to correlate with the results of a bioassay.
  - **Physical durability**, a measure of the ability of the product to resist physical damage or degradation under real-world use. May be measured through monitoring products post-deployment or by artificially recreating the conditions of real-world use, for example the use of standardised washing methods to mimic the use of ITNs.
  - **Resistance monitoring**, which should include a defined discriminating dose and method of exposure to monitor for the decreased sensitivity of the target species to the AI/s.
- **Interaction with existing tools or AIs:** vector control tools do not exist in isolation and multiple tools which may be deployed in an integrated manner or inadvertently be used together in the same location.
  - It is important to understand how the effectiveness of a new product may affect or be affected by other tools which may be used in the same location, for example an emanator with a repellent effect may reduce interaction of mosquitoes with an ITN and reduce its killing effect, or a synergist on an ITN may reduce the lethal effect of a pro-insecticide in an IRS formulation.
  - The cross-resistance risk needs to be considered for a new AI deployed in an area where resistance mechanisms already exist in the vector population as a result of exposure to other AIs.

Although the focus of this Editorial has been on adulticides against *Anopheles*, many of the same principles apply to mosquito control tools more generally and against a wider range of insect vectors. There is a lack of specific guidance on the evaluation of products targeting *Aedes* mosquitoes, partly due to the fact that the available methods to monitor *Aedes* populations are insufficient, and so measuring entomological impact is difficult. Products or control efforts aimed at *Aedes* are also very rarely used in isolation, necessitating the evaluation of integrated approaches, and making the link between bioassay results and predicted impact more complicated. It is a similar story for products used in larval control and for commercial products such as emanators or spatial sprays as well as newer classes under evaluation. 

The pipeline for new vector control tools has never been richer, with a variety of product types and vector control strategies under evaluation for both epidemiological and entomological impact. This pipeline is an achievement to be celebrated, but all of these approaches will require testing methods to measure their efficacy and predict or directly determine entomological and epidemiological impact. The same rigorous approach can be applied to other phases of product development, for example in the screening of new AIs or in formulation development. In all these areas we recommend that the same considerations be taken in developing and validating the required standardised testing methods, including clearly defining the relevant endpoints, standardising or characterising inputs and testing parameters, and being clear on how to analyse, interpret and report data in order to use the results to make robust, evidence-based decisions.
